# Immunogenic cell death triggered by impaired deubiquitination in multiple myeloma relies on dysregulated type I interferon signaling

**DOI:** 10.3389/fimmu.2023.982720

**Published:** 2023-03-02

**Authors:** Zeinab Waad Sadiq, Annamaria Brioli, Ruba Al-Abdulla, Gonca Çetin, Jacqueline Schütt, Hugo Murua Escobar, Elke Krüger, Frédéric Ebstein

**Affiliations:** ^1^ Institut für Medizinische Biochemie und Molekularbiologie (IMBM), Universitätsmedizin Greifswald, Greifswald, Germany; ^2^ Klinik und Poliklinik für Innere Medizin C, Universitätsmedizin Greifswald, Greifswald, Germany; ^3^ Klinik für Innere Medizin II, Universitätsklinikum Jena, Jena, Germany; ^4^ Department of Medicine, Clinic III, Hematology, Oncology, Palliative Medicine, Rostock University Medical Center, Rostock, Germany

**Keywords:** ubiquitin, proteasome, interferon, unfolded protein response, integrated stress response

## Abstract

**Introduction:**

Proteasome inhibition is first line therapy in multiple myeloma (MM). The immunological potential of cell death triggered by defects of the ubiquitin-proteasome system (UPS) and subsequent perturbations of protein homeostasis is, however, less well defined.

**Methods:**

In this paper, we applied the protein homeostasis disruptors bortezomib (BTZ), ONX0914, RA190 and PR619 to various MM cell lines and primary patient samples to investigate their ability to induce immunogenic cell death (ICD).

**Results:**

Our data show that while BTZ treatment triggers sterile type I interferon (IFN) responses, exposure of the cells to ONX0914 or RA190 was mostly immunologically silent. Interestingly, inhibition of protein de-ubiquitination by PR619 was associated with the acquisition of a strong type I IFN gene signature which relied on key components of the unfolded protein and integrated stress responses including inositol-requiring enzyme 1 (IRE1), protein kinase R (PKR) and general control nonderepressible 2 (GCN2). The immunological relevance of blocking de-ubiquitination in MM was further reflected by the ability of PR619-induced apoptotic cells to facilitate dendritic cell (DC) maturation *via* type I IFN-dependent mechanisms.

**Conclusion:**

Altogether, our findings identify de-ubiquitination inhibition as a promising strategy for inducing ICD of MM to expand current available treatments.

## 1 Introduction

One major peculiarity of cancer is its inherent ability to escape immune surveillance. The mechanisms by which tumor cells hijack the immune system are diverse but mostly rely on the cumulative acquisition of genomic changes that lead to a progressive decline of their immunogenicity (1, 2). Typical and prominent features of poorly immunogenic tumor cells include (i) low expression of HLA class I molecules and/or co-stimulatory molecules (3, 4), (ii) inefficient antigen processing (5–7), (iii) tryptophan depletion (8, 9), (iv) upregulation of checkpoint inhibitors (10), (v) increased production of anti-inflammatory mediators (11, 12) and (vi) resistance to killing by cytotoxic T cells (CTL) (13, 14) among others.

Strategies aimed at increasing tumor immunogenicity have long been investigated in the context of dendritic cells (DC)-based cancer vaccine development (15–17). Indeed, due to their unique ability to stimulate T cells (18, 19), DC represent attractive vectors used in active antitumor immunotherapy (20, 21). One route to supply DC with a broad spectrum of tumor-associated antigens is to feed them with killed autologous tumor cells (22–25). In this process, a wide array of tumor antigens is taken up by DC and gains access to the HLA class I and II presentation pathways for subsequent priming of naïve CD8+ and CD4+ T cells (26, 27). The process of DC maturation, an important requirement for the initiation of primary immune responses, is characterized by the upregulation of CD86, CD80, CD83, and CD40 costimulatory molecules as well as the secretion of IL-12 and IL-10 (28–31). DC maturation is typically achieved following exposure to so-called “danger signals” including pathogen-associated molecular patterns (PAMP) such as lipopolysaccharide (LPS) and foreign nucleic acids (32, 33). Depending on the immunogenicity of the cells used for DC loading, DC maturation may be positively or negatively affected. Early studies have shown that the uptake of necrotic cells and/or cell lysates by DC favor their maturation *via* uncontrolled release of damage-associated molecular patterns (DAMP) (34–36). By contrast, the removal of physiologically occurring apoptotic bodies by DC (a process called efferocytosis) seems to exert detrimental effects on the DC maturation process even in the presence of PAMP (37–39), thereby contributing to peripheral tolerance (40–42). Nonetheless, depending on the stimulus and/or the conditions under which cell death is induced, apoptosis may become an immunogenic process supporting DC maturation. Herein, viral-infected and heat shock-stressed apoptotic cells have been shown to promote potent primary immune responses (43–47). These studies have brought the concept of immunogenic cell death (ICD) which itself is defined by the ability of dying cells to deliver immunostimulatory signals promoting DC maturation (48). Over the last two decades, an increasing number of ICD-inducing agents have been identified including anthracyclines, big potassium (BK) channel agonists as well as endoplasmic reticulum (ER) stress-inducing agents (49–52).

In multiple myeloma (MM), the second most frequent hematological cancer, proteasome inhibitors were recently shown to induce ICD (53, 54). Proteasomes are key components of the ubiquitin-proteasome system (UPS), a complex biochemical process which ensures the breakdown of ubiquitin-marked proteins into peptides (55–57). Given its fundamental role in the regulation of protein homeostasis, the UPS represents a particularly vulnerable pathway whose dysfunction may rapidly compromise cell viability (58). The causal relationship between proteasome inhibition and ICD supports the growing consensus that proteasome defects lead to autoinflammation (59–61). It remains however unclear whether the acquisition of immunogenicity under these conditions is an immediate and specific effect of proteasome inhibition or a more distant consequence of overall proteostatic perturbation. A better understanding of these processes is of highly clinical relevance, as MM is still an incurable disease, and resistance to proteasome inhibition invariably occurs. To address this point, we have investigated various protein homeostasis disruptors for their ability to induce ICD in MM cell lines. We show that inhibition of de-ubiquitination by PR619 induces ICD and facilitates DC maturation by activation of type I IFN signaling *via* signal transducers of the integrated stress response (ISR) and the unfolded protein response (UPR). Altogether, these findings support the notion that proteome perturbations confer immunogenicity to MM by delivering danger signals which are integrated by the UPR and ISR.

## 2 Materials and methods

### 2.1 Cell lines and culture conditions

The monocytic cell line THP-1 and the MM cell lines RPMI-8226, RPMI-R5, MM1S, U266 and OPM-2 were cultivated in standard RPMI1640 with 2 mM stable glutamine and supplemented with 10% FBS and 1% penicillin/streptomycin. The MM cell line KMS12BM was cultivated in standard RPMI1640 with 2 mM stable glutamine and supplemented with 20% FBS and 1% penicillin/streptomycin. The MM cell line NCI-H929 was cultivated in standard RPMI1640 with 2 mM stable glutamine and supplemented with 20% FBS, 1% penicillin/streptomycin and 1 mM sodium pyruvate. The HS5 stromal and SH-SY5Y neuroblastoma cell lines were cultivated in DMEM supplemented with 10% FBS and 1% penicillin/streptomycin. T lymphocytes were expanded from Ficoll-enriched PBMC isolated from a healthy donor using PHA-L, IL-2 and feeder cells, as previously described (62). Dendritic cells (DC) were generated from monocytes isolated from heathy donors and were kept in culture for 5 days using RPMI1640 in the presence of 500 U/mL GM-CSF and 50 U/mL IL-4 (both purchased from Miltenyi Biotec), as previously described (63). At day 5, suspension DC were collected together with adherent DC which were detached from the flask by incubating them with PBS/EDTA (2 mM) for 20 min at 37°C. Day 5-immature DC were plated on 24-well plates (1×10^6^ cells/well) and co-cultured with apoptotic NCI-H929 cells (at a ratio 1:2) in a final volume of 2 mL. In some experiments, DC fed with apoptotic NCI-H929 cells were cultivated with 10 µg/ml of anti-IFNAR2 neutralizing antibody (clone MMHAR-2) purchased from R&D Systems. Primary CD138^+^ MM cells were isolated from bone marrow aspirates of two patients (MM14 and MM90) with relapsed MM using Ficoll density gradient centrifugation and manual magnetic cell sorting (CD138 MicroBeads, Miltenyi Biotech GmbH), as previously described (64). Multiple myeloma sample collection was approved by the Review Board and both patients provided written informed consent (Ethic number 2018-1157-Material).

### 2.2 Chemical reagents

Bortezomib (BTZ) was kindly provided by Prof. Christian Andreas Schmidt (Internal Medicine C, University Medicine Greifswald). The ONX0914 (PR-957), PR619 and ISRIB organic compounds were purchased from Absource Diagnostics GmbH (Munich, Germany). The small-molecule inhibitors RA190, H-151, 4µ8C, C16 and Guanabenz targeting ADRM1/Rpn13, STING, IRE1α, PKR and GADD34, respectively were purchased from Merck Millipore. The TLR3/double strand RNA antagonist was a product from Merck Millipore as well. The BX795 and A-92 compound inhibiting TBK1 and GCN2 were products from Axon Medchem. The JAK1/2 small-molecule inhibitor Baricitinib was from MedChemExpress. Tunicamycin (T7765) was purchased from Merck.

### 2.3 MTT assay

MM cell lines were seeded on flat bottom 96-well plates at 5.10^6^ cells/mL with increasing concentrations of BTZ, ONX0914, RA190 and PR619. At 24-hour post-treatment, 0.85 mg/mL of (3-(4,5-Dimethylthiazol-2-yl)-2,5-diphenyltetrazolium bromide (MTT, thiazolyl blue, Carl Roth) solution was added to the cells for 3 h at 37°C. Dissolution of formed crystalline was achieved by adding 100 µL 10% SDS to the cell suspension. After incubation overnight, absorbance was measured at 562 nm on a plate reader.

### 2.4 RNA extraction and qPCR

Total RNA was isolated from snap-frozen cell pellets using the innue prep RNA minikit from Analytic Jena AG following the manufacturer’s recommendations. Five hundred nanograms of total RNA were then used for cDNA synthesis using the M-MLV reverse transcriptase (Promega). Quantitative real-time PCR was conducted in duplicates using the TB Green Premix Ex Taq from Takara Bio together with primers specific for IFI27, IFI44L, IFIT1, ISG15, RSAD2, IFI44, MX1, SIGLEC1, TNFA, IL1B, IL6, IL24 and RPLP0 and/or GAPDH. relative changes in gene expression was analyzed using 2(-Delta Delta C(T)) method and RPLP0 and/or GAPDH as housekeeping genes.

### 2.5 SDS-PAGE and western-blotting

Snap-frozen cell pellets were lysed in standard RIPA buffer (50 mM Tris pH 7.5, 150 mM NaCl, 2 mM EDTA, 1 mM N-ethylmaleimide, 10 µM MG-132, 1% NP40, 0.1% SDS) and protein lysates were quantified by BCA (Thermofisher) following the manufacturer’s instructions. Ten to forty micrograms of total protein were separated by 10 or 12.5% SDS-PAGE and subsequently blotted onto PVDF membranes using a standard wet blot transfer procedure (200V for 1h). After a 20-min incubation with 1X Roti^®^-Block (Carl Roth^®^) at room temperature, membranes were incubated overnight at 4°C with primary antibodies specific for β1 (clone MCP421), β2 (clone MCP165), α6 (clone MCP20), ubiquitin (clone FK2) all purchased from Enzo Life Sciences, Inc. Other primary antibodies include anti-TCF11 (clone D5B10), anti-PERK (clone C33E10), anti-(p)PERK (#3179), anti-IRE1α (#3294), anti-ATF6 (clone D4Z8V), anti-PKR (1297), anti-GCN2 (65981), anti-eIF2α (9722), anti-(p)eiF2α (9721), anti-4E-BP1 (clone 53H11), anti-(p)4E-BP1 (2855s), anti-GAPDH (clone 14C10), anti-caspase-3 (9662S), anti-cleaved caspase-3 (9661L), anti-TBK1 (3013), anti-(p)TBK1 (clone D52C2), anti-IRF3 (4302), anti-STAT1 (clone 2x) and anti-(p)STAT1 (clone 58D6) all products from Cell Signaling Technology. Antibodies directed against (p)PKR (ab226852), α-tubulin (clone DM1A) and β5 (ab3330) were obtained from Abcam. Antibodies specific for (p)IRE1 (PA1-16927), β2i/MECL1 (PA5-19146) and (p)IRF3 (PA5-38285) were from Thermofisher. The monoclonal antibody directed against β5i (clone A-12), was a product from Santa Cruz Biotechnology, Inc. The β1i/LMP2 antiserum (K221) was a laboratory stock already described elsewhere (65). The antibody specific for (p)GCN2 (AF7605-SP) was from R&D systems. After incubation, membranes were washed three times with PBS/0.2% Tween and incubated for 1 h at RT with anti-mouse or –rabbit HRP conjugated secondary antibodies (1/5.000). Proteins were visualized using an enhanced chemiluminescence detection kit (ECL) (Biorad). The ImageJ 1.48v software was used for densitometry analysis of the ECL signals.

### 2.6 Flow cytometry

Dendritic cells were washed twice with PBS and resuspended in PBS/1% BSA with primary antibodies at 4°C for 20 min and resuspended in PBS for phenotypical analyses. Flow cytometry was performed with a MACSQuant10 flow cytometer (Miltenyi Biotec) and data were analyzed with MACSQuantify™ software. APC- or PE-conjugated monoclonal antibodies (all from Miltenyi Biotec) against CD80 (clone 2D10), CD83 (clone HB15) and CD86 (clone FM95) were used for phenotypic analysis. Flow cytometry was also used to measure calreticulin (CRL) cell surface expression on NCI-H929 cells exposed to DMSO, BTZ, ONX0914, RA-190 or PR-619 for 24 and 48h using a PE-conjugated anti-CRL primary antibody (clone FMC-75) from Enzo Life Sciences.

### 2.7 Measurement of ATP release

Supernatants from NCI-H929 cells exposed to DMSO, BTZ, ONX0914, RA-190 or PR-619 were collected after 6 or 24h of treatment and assessed for their ATP content using the RealTime-Glo™ Extracellular ATP Assay from Promega following the manufacturer’s recommendations.

### 2.8 Data representation and statistical analyses

Data are typically median or mean ± SEM from at least three independent experiments and analyzed by paired t-test between two groups. All charts and statistical analyses were generated using GraphPad Prism version 8. A *p*-value <0.05 was considered significant. All raw data are available on request from authors.

## 3 Results

### 3.1 The protein homeostasis disruptors BTZ, ONX014, RA190, and PR619 differ in their antitumor activities against MM

To deepen the relevance of disrupting protein homeostasis as a targeted strategy for MM treatment, we first examined the anti-proliferative effects of four pharmacological agents interfering with the protein homeostasis network at different levels. Compounds used in this study included small-molecule proteasome and immunoproteasome inhibitors, namely bortezomib (BTZ) and ONX-0914 targeting the β5/β5i and β5i catalytic proteasome subunits, respectively (66, 67); RA190 which blocks ubiquitin recognition by the proteasomal ubiquitin receptor RPN13/ADRM1 (68) as well as PR619, a permeable pan-inhibitor of deubiquitinating enzymes (DUB) (69). Among these compounds, only BTZ is currently in clinical use for the treatment of MM, although ONX0914 and RA190 already showed activity in preclinical models (70). As shown in **Figure S1**, BTZ treatment successfully compromised cell growth in RPMI-8226, RPMI-R5, MM1S, NCI-H929 and KMS12BM cell lines. In line with previous studies (71, 72), OPM-2 and U266 cells were resistant to BTZ with approximately only half of the cells dying at 50 µM (**Figure S1**). Immunoproteasome inhibition by ONX0914 showed a toxicity profile which was quite similar to that of BTZ, although higher concentrations were required to compromise cell viability and had only marginal effects on the viability of the OPM-2 and U266 cell lines (**Figure S1**). By contrast, RA190 efficiently induced cell death in all seven investigated MM cells within 24 h of treatment albeit to a lesser extent in the OPM-2 and U266 MM cell lines which became only sensitive from concentrations >10 µM (**Figure S1**). Likewise, exposing MM cells to PR619 resulted in impaired cell growth in all seven tested cell lines at concentrations down to 6 µM except for the OPM-2 and U266 cell lines which were less sensitive and required a minimal concentration of 10 µM (**Figure S1**).

### 3.2 MM cells are equipped mostly with standard proteasomes and/or β5i/β1/β2 mixed type proteasomes

In view of the heterogeneous sensitivity of MM cells to BTZ and/or ONX0914 treatments, we next sought to determine proteasome composition in the seven investigated MM cell lines. As shown in **Figures S2**, **S3**, all MM cell lines expressed standard proteasomes, as evidenced by constitutive expression of the β1, β2 and β5 standard subunits. From the three immunoproteasome subunits, only β5i was found to be consistently expressed across nearly all MM samples with the exception of the OPM-2 cell line, which exclusively contained standard proteasomes (**Figures S2**, **S3**). These data thus indicate that most MM cells were endowed with standard proteasomes and/or mixed-type proteasomes carrying the β5i inducible subunit together with β1 and β2 standard subunits. Besides, these findings further suggest that resistance to proteasome inhibition observed in the OPM-2 and U266 cells may be caused by reduced β5i expression and proteasome amounts, respectively. This assumption is supported by previous studies indicating that β5i can restore BTZ sensitivity (73).

### 3.3 The protein homeostasis disruptors BTZ, ONX0914, RA190, and PR619 vary in their ability to initiate sterile type I IFN responses in MM

**Figure 1:**
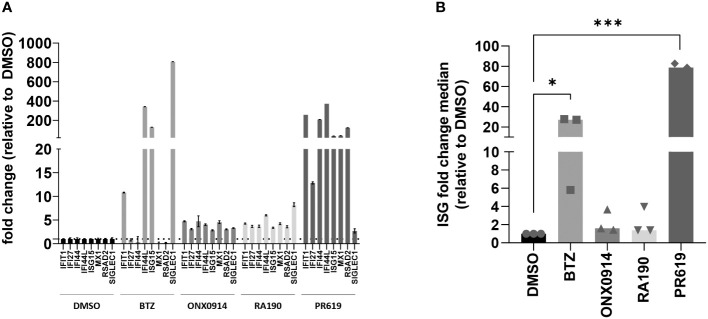
Analysis of the IFN-stimulated gene (ISG) expression profile in NCI-H929 MM cells exposed to protein homeostasis disruptors. **(A)** Gene expression of eight typical IFN-stimulated genes (*IFIT1*, *IFI27*, *IFI44*, *IFI44L*, *ISG15*, *MX1*, *RSAD2* and *SIGLEC1*) was assayed by RT-qPCR on NCI-H929 MM cell lines after a 12-h exposure to BTZ, ONX0914, RA190, PR619 or DMSO (control), as indicated. Expression levels were normalized to housekeeping genes (RPLP0) and relative quantifications (RQ) are presented as fold change over cells exposed to DMSO. Shown is one representative experiment out of three. **(B)** Shown are fold change median values of the eight ISG over DMSO measured in three independent experiments. Statistical significance was assessed by paired t test (*p<0.05, *** *p*<0.001).

Because rare proteasome loss-of-function mutations typically cause type I interferonopathies (59, 60, 74), we next aimed to determine whether protein homeostasis disruption triggered by BTZ, ONX0914, RA190 or PR619 was associated with the acquisition of a type I IFN gene signature in MM. To this end, the transcription rate of eight typical IFN-stimulated genes (ISG) (i.e. *IFIT1*, *IFI27*, *IFI44*, *IFI44L*, *ISG15*, *MX1*, *RSAD2* and *SIGLEC1*) was evaluated in MM cells at 12-h post-treatment by qPCR. As shown in **Figures 1**, **S4**, BTZ treatment resulted in the initiation of a type I IFN response in all cell lines except OPM-2. By contrast, all seven investigated MM cells failed to generate a type I IFN signature in response to RA190, as evidenced by unchanged ISG scores after treatment (**Figures 1**, **S4**). Likewise, immunoproteasome inhibition by ONX0914 was immunologically silent across all MM cell lines **(** **Figures 1**, **S4**). Interestingly, blocking DUB activity by PR619 was immunostimulant in only one cell line, namely NCI-H929 in which the ISG fold change median was >100-fold higher relative to untreated cells. To further explore the responsiveness of MM to DUB inhibition, two additional primary samples (MM14 and MM90) from myeloma patients as first relapse were tested for their capacity of inducing ISG upon PR619 treatment. As shown in **Figure S5**, while MM90 cells generated a type I IFN signature in response to PR619, MM14 cells failed to do so –in spite of a moderate upregulation of the *IFIT1* and *RSAD2* genes. Taken together, these data demonstrate that the ability of MM to mount a type I IFN response upon protein homeostasis disruption is largely dependent on both stimulus and cell characteristics.

### 3.4 Both UPR and ISR are constitutively activated in NCI-H929 MM cells

**Figure 2:**
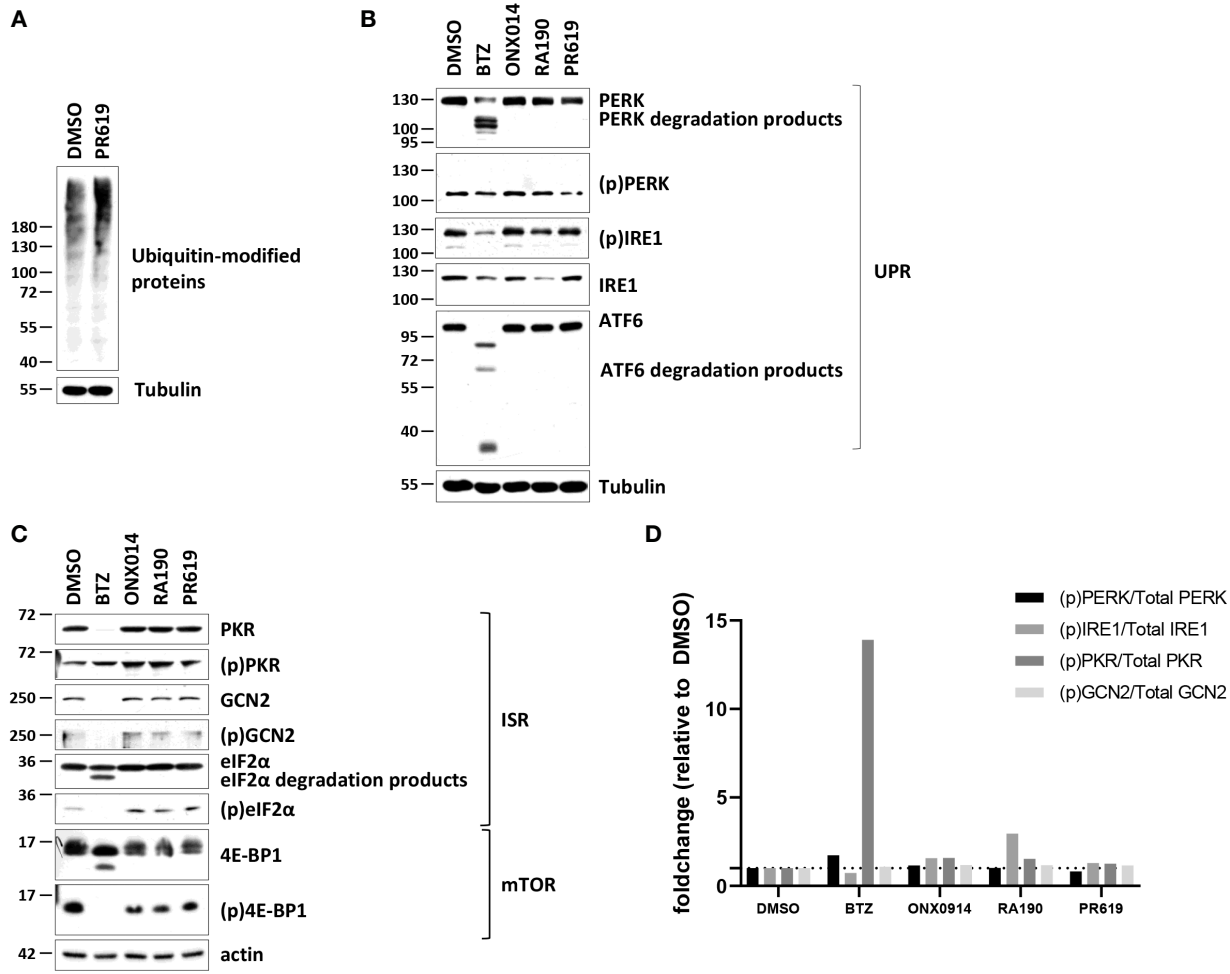
Western-blot analysis of the ubiquitin, ISR and UPR expression profiles in NCI-H929 cells exposed to BTZ, ONX-0914, RA190 or PR619. **(A)** NCI-H929 exposed to PR619 (1,5 µM) or left untreated were subjected to protein extraction and subsequent SDS-PAGE/western blotting using antibodies specific for ubiquitin and actin (loading control), as indicated. Shown is one representative experiment out of three. **(B)** Equal amounts of protein lysates derived from NCI-H929 exposed to a 12-h treatment with DMSO, BTZ (50 nM), ONX-0914 (50 nM), RA190 (50 nM) or PR619 (1,5 µM) were analyzed by SDS-PAGE/western-blotting using antibodies directed against PKR, (p)PKR, GCN2, (p)GCN2, eIF2α, (p)eIF2α, 4E-BP1, (p)4E-BP1 and tubulin (loading control), as indicated. Shown is one representative experiment out of three. **(C)** NCI-H929 whole cell-lysates described in **(B)** were further assessed for their contents in PERK, (p)PERK, IRE1, (p)IRE1, ATF6 by SDS-PAGE/western-blotting, as indicated. Equal protein loading was ensured by probing the membranes with an actin antibody. Shown is one representative experiment out of three. **(D)** Densitometric analysis of phosphorylated of PERK, IRE1, PKR and GCN2 normalized to total proteins and reported as foldchange relative to DMSO.

The observation that PR619 treatment results in the generation of a type I IFN gene signature in NCI-H929 cells is interesting and raises the question as to how the loss of DUB activity is sensed as a “danger signal” in these cells. As shown in **Figures 2A**, **S6**, PR619 treatment led to an increased accumulation of ubiquitin-modified proteins in NCI-H929 cells, thereby confirming that impairment of the protein de-ubiquitination process was associated with perturbations of the whole-cell proteome under these conditions.

Intracellular protein homeostasis is typically surveilled by the signaling arms of the UPR IRE1α, ATF6 and PERK within the ER membrane (75). The activation/phosphorylation status of IRE1α and PERK was therefore next assessed in NCI-H929 cells subjected to a 12-hour treatment of BTZ, ONX0914, RA190 or PR619. Consistent with the notion that the UPR is constitutively active in MM (76), the phosphorylated (p) forms of IRE1α and PERK were already highly expressed in untreated NCI-H929 cells and remained unchanged following exposure to ONX0914, RA190 or PR619 **(** **Figures 2B**, **S6**). Strikingly, treating the cells with BTZ resulted in a drop of the unmodified forms of IRE1α and PERK (**Figures 2B**, **S6**). Likewise, the expression levels of (p)IRE1α, ATF6 and, to a lesser extent, (p)PERK were reduced in BTZ-treated cells (**Figures 2B**, **S6**). Our densitometry analysis of the signals revealed that the relation of the phosphorylated fractions of PERK and IRE1α to the total ones did not vary in BTZ-treated cells (**Figure 2D**), thereby indicating that UPR activity remained constant during the course of BTZ treatment.

Other pathways in charge of monitoring intracellular protein homeostasis include ISR which is typically initiated by the GCN2 and/or PKR kinases upon amino acid depletion and proteotoxic stress, respectively (77, 78). Interestingly, NCI-H929 cells treated with BTZ, ONX0914, RA190 or PR619 exhibited reduced mTORC1 signaling, as evidenced by decreased phosphorylation of mTOR downstream target (p)4E-BP1 (**Figures 2C**, **S6**) Since mTORC1 senses free amino acids (79), these data suggest that these cells may suffer from amino acid deficiency. However, no discernable differences could be observed in the expression level of (p)GCN2 following treatment (**Figures 2C**, **S6** **)**. In a similar fashion to IRE1α and PERK, GCN2 was downregulated upon BTZ exposure **(** **Figures 2C**, **S6**). Like GCN2 and PERK, PKR was constitutively phosphorylated in NCI-H929 cells and the (p)PKR/total PKR ratio was increased only following BTZ treatment (**Figures 2C, D,** **S6**). Nevertheless, BTZ failed to promote eIF2α phosphorylation whose expression levels even declined when compared to those from control cells **(** **Figures 2C**, **S6** **)**. This observation is likely related to the fact that BTZ-treated NCI-H929 cells may undergo apoptosis, as evidenced by increased breakdown of unmodified eIF2α **(** **Figures 2C**, **S6** **)**. Exposing the cells to ER stress inducer tunicamycin did not result in increased phosphorylation of the IRE1α, PERK and GCN2 proteins (**Figure S7**), confirming that UPR and ISR activities in these cells already reached their maximal levels even under basal conditions.

### 3.5 The type I IFN gene signature mediated by the loss of DUB activity in PR619-treated NCI-H929 cells relies on both the UPR and ISR

We next sought to determine the molecular mechanisms by which pharmacological inhibition of protein de-ubiquitination by PR619 promotes the acquisition of a type I IFN gene signature by NCI-H929 cells. Typically, type I IFN responses are initiated during viral infections in response to foreign nucleic acids such as double-stranded (ds)RNA or cytosine-phosphate-guanosine (CpG) motifs which are sensed by specialized endosomal and/or cytosolic receptors (80). Engagement of such DNA/RNA sensors triggers a signaling cascade ultimately resulting in the TBK1-mediated phosphorylation of the transcription factor IRF3 which subsequently translocates into the nucleus to induce the synthesis of IFN-α/β (81). Once released and bound to its receptor, IFN-α/β triggers a JAK/STAT signaling pathway resulting in the transcription of ISG. Other potent inducers of type I IFN include sterile danger signals such as mitochondrial and nuclear nucleic acid leakage as well as cytosolic IL-24 (58, 82, 83).

**Figure 3:**
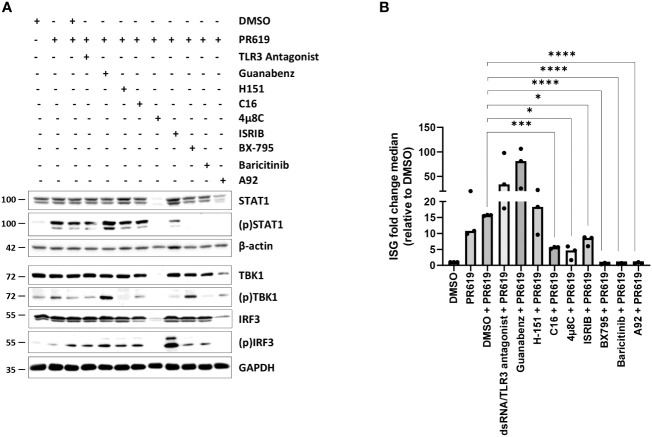
Effects of various signaling pathway small-molecule inhibitors on the type I IFN gene signature triggered by PR619 in NCI-H929 cells. **(A)** NCI-H929 cells were exposed to DMSO, TLR3/ds RNA antagonist (100 µM), guanabenz (50 µM), H-151 (2 µM), C16 (1 µM), 4µ8C (100 µM), ISRIB (200 nM), BX795 (1 µM), baricitinib (1 µM) or A92 (10 µM) for 2 hours prior to an overnight treatment with DMSO or PR619 (1,5 µM), as indicated. Samples were collected and assessed for their contents in STAT1, (p)STAT1, TBK1, (p)TBK1, IRF3 and (p)IRF3 by SDS-PAGE/western-blotting, as indicated. Equal protein loading was verified by probing the membrane with monoclonal antibodies specific for β-actin or GAPDH. Shown is one representative experiment out of three. **(B)** NCI-H929 samples described in **(A)** were subjected to RNA extraction and subsequent RT-qPCR analysis for the eight *IFI27*, *IFIT1*, *IFI44*, *IFI44L*, *ISG15*, *RSAD2*, *MX1* and *SIGLEC1* genes. Shown are fold change median values of the eight ISG over DMSO measured in three independent experiments. Statistical significance was assessed by ratio paired t test (*p<0.05, *** *p*<0.001, ****p<0.0001).

As shown in **Figures 3A, B**, **S8**, **S9**, blocking TBK1 by BX795 in PR619-treated cells resulted in a strong ISG downregulation as a consequence of reduced levels of (p)IRF3 and (p)STAT1. Similarly, baricitinib completely abolished STAT1 phosphorylation and ISG transcription in response to PR619 (**Figures 3A, B**, **S8**, **S9**). These data indicate that the ISG signature induced by PR619 follows a two-step process in which IFN-α/β is first synthetized and then secreted to act in an autocrine/paracrine fashion. Our data further suggest that type I IFN under these conditions is not driven by host nucleic acids cells, since inhibition of the nucleic acid receptors TLR3 and STING by dsRNA/TLR3 antagonist and H-151, respectively had no discernable impact on STAT1 phosphorylation and/or ISG expression profile **(** **Figures 3B**, **S8**, **S9** **)**. Given the described ability of the UPR/ISR to induce sterile inflammation (82), we next asked whether it participated in the PR619-mediated type I IFN response in NCI-H929 cells. To address this point, we took advantage of commercially available inhibitors targeting the UPR/ISR at different levels including 4µ8C, C16, A92 and guanabenz which inhibit IRE1α, PKR, GCN2 and eIF2α dephosphorylation, respectively (84–86). As illustrated in **Figures 3B**, **S8**, **S9**, while C16, 4μ8C and A92 substantially suppressed ISG upregulation in PR619-treated cells, guanabenz slightly exacerbated it. These data indicate that PKR, IRE1α, and GCN2 were involved in the PR619-mediated type I IFN gene signature. Interestingly, although both C16 and A92 exerted a suppressive activity, only A92 resulted in decreased expression of (p)STAT1, suggesting that the type I IFN response induced by PKR was at least partially STAT1-independent. Exposing the cells to the organic compound ISRIB which antagonizes the (p)eIF2α-induced translation arrest by increasing eIF2B levels (87–89) showed less pronounced inhibitory effects on ISG induction in response to PR619 (**Figures 3B**, **S8**, **S9**), indicating that the shutdown of protein synthesis is only partially involved in this process.

### 3.6 Protein homeostasis disruption caused by PR619 induces ICD

**Figure 4:**
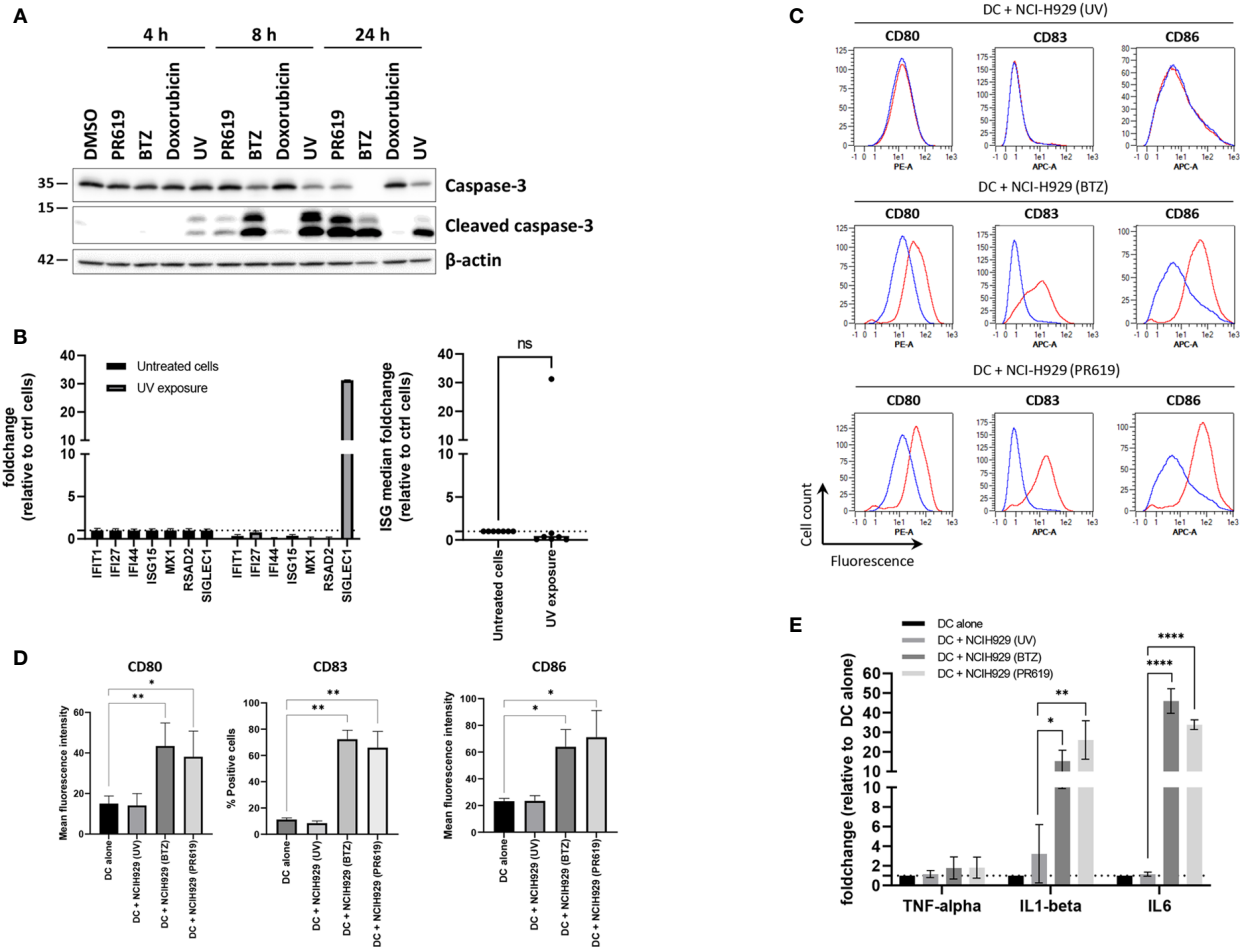
Apoptotic NCI-H929 cells induced by PR619 promote spontaneous DC maturation. **(A)** NCI-H929 cells were treated with PR619, BTZ, doxorubicin or exposed to UV-B irradiation prior to protein extraction and SDS-PAGE/western-blot analysis using antibodies specific for caspase-3 and cleaved caspase-3, as indicated. Shown is one representative experiment out of three **(B)** NCI-H929 cells subjected to UV-B exposure were compared to untreated (control) cells and assessed for expression of ISG transcripts (*IFI27*, *IFIT1*, *IFI44*, *ISG15*, *RSAD2*, *MX1* and *SIGLEC1*) by RT-qPCR, as indicated. Expression levels were normalized to housekeeping genes and relative quantifications (RQ) are presented as fold change (left) and fold change median values (right) over untreated cells, as indicated. **(C)** Representative histogram overlays of flow cytometry analysis of DC for cell surface expression of DC maturation markers CD80, CD83 and CD86 following a 24-h co-culture with UV-, BTZ- or PR619-induced NCI-H929, as indicated. The results obtained with DC w/o co-culture and with co-culture are indicated by the blue and red lines, respectively. **(D)** Variations of the percentage or the mean fluorescence intensity of the DC maturation markers following co-culture with UV-, BTZ- or PR619-treated NCI-H929 cells, as indicated. Shown are the means and SEM calculated from four independent experiments. Statistical significance was assessed by ratio paired t test where *indicates *p*<0.05 and ** indicates *p*<0.01. **(E)** DC alone or cultured with UV-, BTZ- or PR619-treated NCI-H929 cells were subjected to RNA extraction and assessed for their content in TNFα, IL1β and IL6 transcripts by RT-qPCR, as indicated. Shown are the means and SEM obtained from four independent experiments. Statistical significance was assessed by ratio paired t test (*p<0.05, **p<0.01, ****p<0.0001) , ns, not significant.

Given that pharmacological inhibition of protein de-ubiquitination by PR619 results in the generation of a type I IFN signature in NCI-H929 cells, we hypothesized that such gene expression profile would confer immunogenic properties to these cells. Having shown that high concentration of PR619 cause cytotoxic effects (**Figure S1**), we next sought to determine the impact of PR619-induced apoptotic NCI-H929 cells on dendritic cells (DC). To this end, NCI-H929 cells were first treated with 6 µM PR619 and compared to cells exposed to UV-B, BTZ and doxorubicin for their ability to cleave caspase-3 over a 24h period of time. As shown in **Figures 4A** , **S10**, exposing NCI-H929 cells to 6 µM PR619 resulted in caspase-3 cleavage within 24 h to a similar extent as seen with UV-B radiation, indicating that it efficiently triggered apoptotic cell death. Of note, apoptosis induced by PR619 or UV-B was much slower than that triggered by BTZ, as evidenced by lower amounts of cleaved caspase-3 at 8 h post-treatment (**Figures 4A**, **S10**). Strikingly, doxorubicin failed to promote caspase-3 cleavage at all investigated time points, suggesting the existence of drug-resistance mechanism preventing the cells to undergo apoptosis. Importantly, cells exposed to UV-B were unable to upregulate ISG except *SIGLEC1* (**Figure 4B**), thereby confirming that, as opposed to PR619 and BTZ-induced apoptosis, cell death mediated by UV-B exposure was devoid of type I IFN. Importantly, day 5-immature DC exposed to apoptotic NCI-H929 cells produced by BTZ or PR619 treatments expressed higher levels of the surface maturation markers CD80, CD83 and CD86 than those cultivated with dead NCI-H929 cells resulting from UV-B exposure (**Figures 4C, D**). Similarly, DC fed with BTZ- and PR619-treated NCI-H929 cells expressed larger amounts of IL1β and IL6 transcripts than DC cultivated alone or exposed to UV-treated NCI-H929 cells (**Figure 4E**). These data demonstrate that, in contrast to UV-B-induced DNA-damage, disruption of protein homeostasis by BTZ or PR619 triggers immunogenic cell death in NCI-H929 cells.

### 3.7 DC maturation induced by PR619-induced NCI-H929 apoptotic cells partially requires type I IFN signaling

**Figure 5:**
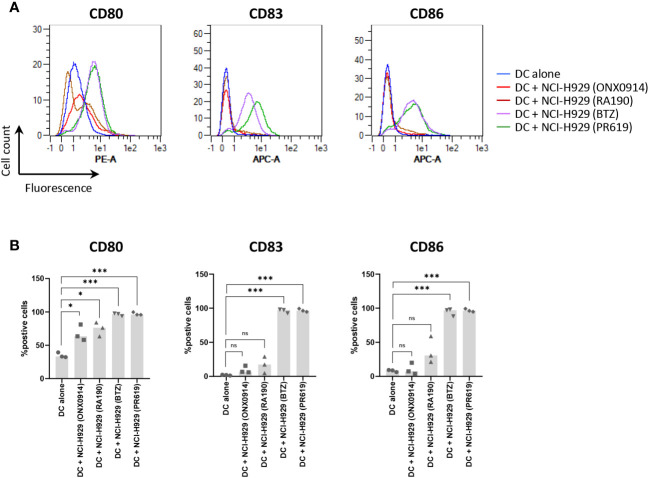
Effects of ONX0914- and RA190-induced cell death on the ability of NCI-H929 cells to deliver stimulatory signals to DC. **(A)** Histogram overlays of flow cytometry analysis of DC cell surface expression of CD80, CD83 and CD86 upon a 24 h-incubation with NCI-H929 dead cells obtained from treatments with ONX0914 (red line), RA190 (brown line), BTZ (purple line) or PR619 (green line), as indicated. Negative control in this experiment consisted of unloaded day 5-immature DC (blue line). Shown is one representative experiment out of three. **(B)** Measurements of the percentage of DC positive for CD80, CD83 or CD86 following co-culture with ONX0914-, RA190-, BTZ- or PR619-induced NCI-H929 apoptotic cells, as indicated. Shown is the median from three independent experiments. Statistical significance was assessed by paired t test where *indicates *p*<0.05 and *** indicates *p*<0.001, ns, not significant.

**Figure 6:**
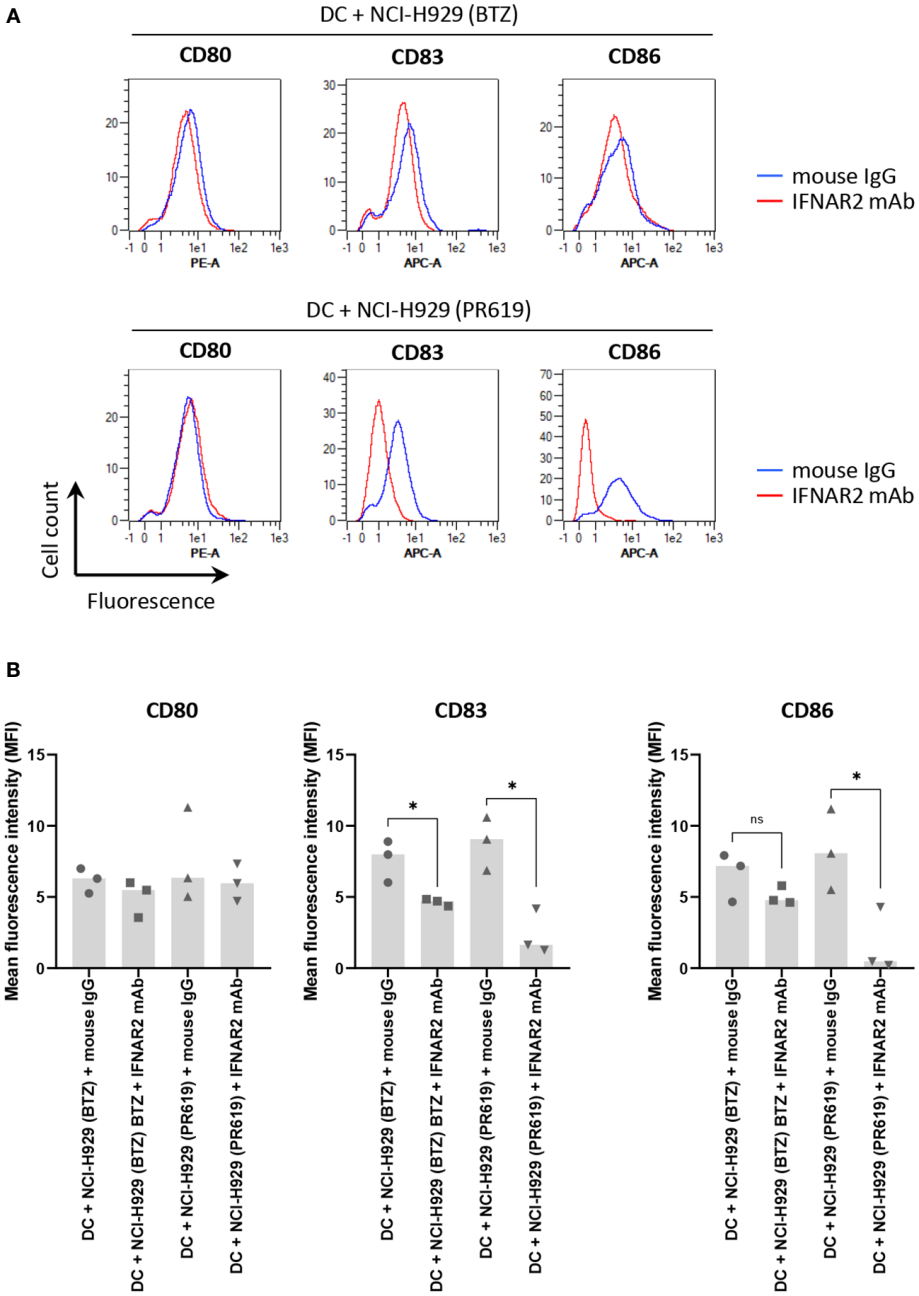
Impact of type I IFN receptor neutralization on the immunogenic properties of BTZ- and PR619-treated NCI-H929 cells exerted on DC. **(A)** Histogram overlays of flow cytometry analysis of CD80, CD83 and CD86 cell surface expression by DC following a 24 h-co-culture with BTZ- or PR619-induced NCI-H929 apoptotic cells in the presence of 10 µg/ml anti-IFNAR2 blocking antibody (red line) or a mouse IgG isotype control (blue line), as indicated. Shown is one representative experiment out of three. **(B)** Measurements of the mean fluorescence intensity (MFI) from DC stained for CD80, CD83 or CD86 cultivated with BTZ- or PR619-induced NCI-H929 apoptotic cells with either mouse IgG isotype control or neutralizing antibody specific for IFNAR2, as indicated. Shown is the median from three independent experiments. Statistical significance was assessed by paired t test where * indicates *p*<0.05 and ns, not significant.

The fact that apoptotic NCI-H929 cells induced by UV-B fail to trigger DC maturation (**Figure 4**) strongly suggests that ICD in these cells is mediated by type I IFN. This assumption is strengthened by the observation that IFN-free apoptotic NCI-H929 cells induced by either ONX0914 or RA190 are unable to fully activate DC (**Figure 5A**). Indeed, the failure of ONX0914- and RA190-induced apoptotic NCI-H929 cells to induce DC maturation was particularly evident when assessing cell surface expression of CD83 and CD86, whose levels remained statistically unchanged when compared to unloaded DC (**Figure 5B**). To further address the role of type I IFN in this process, DC were fed with apoptotic NCI-H929 cells induced by BTZ or PR619 in the presence of anti-IFNAR2 antibodies which neutralize type I IFN receptors. As shown in **Figures 6A, B**, blocking type I IFN signaling by anti-IFNAR2 antibodies resulted in reduced cell surface expression of CD83 and CD86 on DC loaded with BTZ- or PR619-induced apoptotic NCI-H929 cells, as determined by flow cytometry. Interestingly, the anti-IFNAR2 antibody had no substantial impact on CD80 expression, suggesting that the immunogenicity properties of BTZ and PR619 did not entirely rely on their capacity of inducing type I IFN.

### 3.8 Apoptosis induced by BTZ, ONX0914, or PR619 is associated with the supply of ICD-specific biomarkers

**Figure 7:**
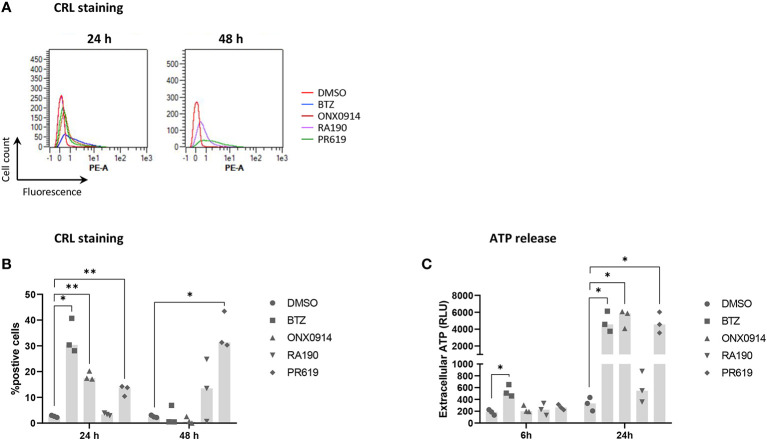
Measurements of calreticulin (CRL) cell surface expression and ATP extracellular release by NCI-H929 cells treated with BTZ, ONX0914, RA190 or PR619. **(A)** Flow cytometry histogram overlays of CRL cell surface expression by NCI-H929 cells following a 24 or 48h-treatment with DMSO (red line), BTZ (blue line), ONX0914 (brown line), RA190 (purple line) or PR619 (green line), as indicated. Shown is one representative experiment out of three. **(B)** Measurements of the percentage of NCI-H929 cells positive for CRL after a 24 or 48 h-incubation with DMSO, BTZ, ONX0914, RA190 or PR619, as indicated. Shown is the median calculated from three independent experiments. Statistical significance was assessed by paired t test where *indicates *p*<0.05 and ** indicates *p*<0.01. **(C)** Bioluminescence analysis of extracellular ATP levels in supernatants from NCI-H929 cells subjected to a 6 or 24 h-treatment with DMSO, BTZ, ONX0914, RA190 or PR619. Shown is the median of the relative light units (RLU) measured following a 5 min-incubation with the luciferase-containing assay medium and calculated from three independent experiments. Statistical significance was assessed by paired t test where * indicates *p*<0.05.

To further characterize the immunogenic potential of cell death triggered by protein homeostasis disruption, NCI-H929 cells were finally tested for their ability to deliver well-established ICD markers in response to BTZ, ONX0914, RA190 or PR619. Herein, NCI-H929 cells induced to apoptosis were monitored for translocation of the ER chaperone protein calreticulin (CRL) to the cell surface, a typical feature of ICD promoting efferocytosis (51, 90). As shown in **Figures 7A, B**, besides RA190, all regimens used in this study led to CRL cell surface expression with different magnitudes and kinetics. Indeed, while BTZ and PR619 treatments allowed 30% of the cells to translocate CRL at 24 and 48h respectively, exposure to ONX0914 resulted in less than 20% CRL-positive cells. Consistently, cell death triggered by RA190 was not accompanied by ATP release (**Figure 7C** **)**, another ICD marker (91–93). This is in sharp contrast to BTZ-, ONX0914- and PR619-treatments of NCI-H929 cells whose supernatants contained large and comparable amounts of extracellular ATP (**Figure 7C**). Altogether, these data indicate that the four protein homeostasis disruptors investigated in this study differed in their ability to deliver danger signals with BTZ, ONX0914 and PR619 triggering ICD, while RA190 remaining immunologically inert.

## 4 Discussion

To date, most strategies aiming at interfering with intracellular protein homeostasis are based on the use of pharmacological agents affecting proteasome function. These include small-molecule inhibitors of the proteasome catalytic subunits such as BTZ, ixazomib or carfilzomib targeting the chymotrypsin-like activity of the β5/β5i subunits and used to treat MM (66, 94). Recently, it was shown that exposure of MM cells to BTZ triggers ICD through the upregulation of type I IFN signaling (53, 54). The observation that proteasome inhibition is associated with type I IFN responses is in line with earlier studies showing that subjects carrying proteasome loss-of-function mutations suffer from type I IFN-driven systemic autoinflammation (95–105). In the present study, we confirm that most MM cell lines acquire a specific type I IFN gene expression profile in response to BTZ (**Figures 1**, **S4**).

Immunoproteasome inhibition by ONX0914 specifically targeting the β5i proteasome catalytic subunit could not promote a type I IFN gene signature in MM cells (**Figures 1**, **S4** **)**. There is controversy in the field with respect to the role of immunoproteasomes in inflammation. A flurry of studies has shown that β5i inhibition by ONX0914 exerts anti-inflammatory effects in autoimmune diseases such as colitis, rheumatoid arthritis (RA), multiple sclerosis (MS) or myocarditis (67, 106–111). Conversely, others have identified *PSMB8* (i.e. β5i) loss-of-function mutations as disease-causing in autoinflammatory syndromes (95–99, 103–105) and *PSMB8* knockout causing elevated inflammation in mouse models of myocarditis (112), Alzheimer’s disease (113) and pancreatitis (114). The reasons for these conflicting data are so far unclear but may reflect distinct consequences between long- and short-term blockage of immunoproteasome function and different experimental set-ups.

Another compound targeting proteasome function is RA190, which in contrast to BTZ and/or ONX0914 does not affect the proteasome chymotrypsin-like activity but the recognition of ubiquitin-modified substrates by proteasomes *via* the ubiquitin receptor ADRM1/Rpn13 (68). In agreement with previous findings (68, 115–117), we could confirm that RA190 exhibits a strong and broad anti-tumor activity against MM including the BTZ-resistant cell lines OPM-2 and U266 (**Figure S1**). However, unlike BTZ, RA190 failed to stimulate MM cells to generate a type I IFN gene signature (**Figures 1**, **S4**). These results are surprising considering that both compounds target the same multi-subunit enzyme. They also suggest that impairment of proteasome activity rather than ubiquitin binding to proteasomes generate sufficient proteotoxic stress to trigger an inflammatory response.

The novelty of this study lies in the observation that blocking protein de-ubiquitination by PR619 induces ICD. Indeed, our data show that PR619 exhibits antiproliferative effects on all seven tested MM cell lines (**Figure S1**). Like BTZ, PR619-induced apoptosis was accompanied by a strong upregulation of ISG in NCI-H929 cells (**Figure 1**). The observation that the acquisition of a type I IFN gene signature upon PR619 treatment was restricted to the NCI-H929 cell line is intriguing. Our investigations on primary MM samples seem to confirm the selective responsiveness of MM to PR619 (**Figure S5**). These results might reflect different DUB expression and/or activity profiles across multiple myeloma(s). Unfortunately, due to the low number of available MM samples, we were not able to associate this selectivity to a specific genetic profile or other disease characteristics. These findings, which need to be confirmed using a larger number of MM samples, support the importance of preliminary molecular profiling to determine the potential of future therapies in precision medicine. This becomes particularly evident in MM in which BTZ is routinely used as first-line therapy, although its ability to confer immunogenicity seems to vary across cell lines (**Figure S4**). Of note, both patients included in the study were treated with BTZ-containing regimens as part of first-line treatment.

Anyhow, the central question arises as to how the loss of DUB activity in NCI-H929 cells leads to sterile type I IFN responses. Interestingly, our data show that the mechanism by which PR619 initiates innate immunity differ from those employed by BTZ. In contrast to BTZ (53, 54), PR619 does not involve the cGAS/STING pathway for the induction of type I IFN gene expression (**Figure 3**). Rather, our small-molecule inhibitor-based experiments support a role for the UPR and ISR in this process. By contrast, the breakdown of critical receptors of the UPR and ISR (i.e. PERK, ATF6, PKR and GCN2) associated with BTZ treatment (**Figures 2**, **S6**, **S7**) did not affect its ability to trigger type I IFN responses (**Figure 1**). Unfortunately, the intrinsic adjuvants effects of interfering RNA molecules (118–120) prevented us to validate the prominent role of the UPR and ISR in the induction of type I IFN by PR619 using gene knockdown strategies. It is further noteworthy that the ISG induction in response to PR619 in these inhibition experiments (**Figure 3**) was lower than that initially observed (**Figure 1**), a variation which is likely to be caused by seeding density differences between the assays. Although the stress sensors IRE1α, PERK, PKR and GCN2 were constitutively activated in MM cells, PR619 treatment was associated with increased phosphorylation of eIF2α (**Figures 2**, **S6**), indicating that the UPR and ISR were engaged under these conditions. As discussed above, ISR activity was required for the ability of NCI-H929 cells to upregulate ISG in response to PR619 (**Figure 3**). Indeed, inhibition of the upstream eIF2α kinases PKR and GCN2 significantly reduced ISG transcription following PR619 treatment (**Figures 3**, **S9**). Accordingly, preventing eIF2α dephosphorylation (84) enhanced the type I IFN response by DUB inhibition (**Figures 3**, **S9**). The ability of PKR, once activated, to engage type I IFN responses is well established (121–124), although the precise signaling cascades remain poorly understood. The signaling pathways that constitutively activate PKR and GCN2 remain unclear but may involve multiple stress stimuli. PKR may be activated by sustained ER stress in a PACT-dependent manner (125, 126), a notion which is in line with the fact that MM are characterized by persistent ER protein homeostasis perturbations due to the high production of immunoglobulins (127). Alternatively, PKR has been recently shown to be stimulated by the aggregation of cytosolic IL-24 (82). Because of persistent ER stress, it is conceivable that MM cells may produce large amounts of IL-24 misfolded species which then accumulate in the cytosol following retro-translocation to activate PKR. Accordingly, it is also tempting to speculate that the failure of the OPM-2 cell line to upregulate ISG in response to BTZ (**Figure S4**) might be caused by the lack of IL-24 expression in these cells (**Figure S11A**). Conversely, IL-24 was strongly expressed in NCI-H929 cells and even further increased following BTZ treatment (**Figure S11B**). It is, however, unlikely that the induction of IL-24 by BTZ is driven by autocrine type I IFN, as NCI-H929 cells do not elevate their IL-24 levels in response to PR619 (**Figure S11B**). This effect seems thus specific to proteasome dysfunction and one could argue that this process also exacerbates autoinflammation by increasing the supply of misfolded IL24 for PKR activation.

In contrast to PKR, the ability of GCN2 to engage type I IFN responses is not well established (121–124). The GCN2 kinase is typically activated by intracellular amino acid shortage (128) but it is unclear whether MM cells suffer from amino acid restriction. This assumption would be, however, consistent with the fact that protein homeostasis is inherently perturbed in MM. Unlike PKR, GCN2 has not been described as a pattern recognition receptor of innate immunity and the mechanisms by which it promotes type I IFN remain unclear. It should be noted that GCN2 substrates are not limited to eIF2α but also include the methionyl-tRNA synthetase (MRS) in response to UV irradiation (129). Interestingly, phosphorylation of MRS facilitates the nuclear translocation of AIMP3 to activate the DNA damage sensors ATM (ataxia-telangiectasia, mutated) and ATR (ATM and Rad3-related) (130) as well as proteasome-mediated degradation of lamin A (131). Of note, lamin A deficiency is associated with genome instability and IFN responses in patients with Hutchinson-Gilford progeria syndrome (132). Whether this pathway is involved in GCN2-dependent type I IFN signature generated in NCI-H929 cells in response to PR619 remains to be determined. Our data further show that beside the ISR, the IRE1α arm of the UPR is involved in the upregulation of ISG induced by PR619 (**Figures 3**, **S9**). Indeed, blocking IRE1α abolished JAK/STAT1 signaling in these cells, as a consequence of reduced STAT1 expression levels (**Figures 3A**, **S8**). Interestingly, IRE1 inhibition in microglia failed to affect STAT1 steady-state expression (133), suggesting that STAT1 regulation by IRE1 is likely to be cell type-dependent.

Importantly, protein homeostasis disruption caused by PR619 was functionally immunogenic, as evidenced by the ability of PR619-induced cell death to facilitate DC maturation *in vitro* (**Figures 4C–E**). Strikingly, blocking type I IFN receptors on DC reduced the ability of PR619-treated cells to activate DC (**Figure 6**), indicating that the immunogenic potential of PR619 vis-à-vis DC was strongly dictated by type I IFN. These findings also indirectly suggest that type I IFN may be newly synthetized released from dying NCI-H929 cells, although we were unable to detect it in their supernatants using luminescent-based ELISA technology (data not shown). The immunostimulatory capacities of PR619-induced apoptotic cells were comparable to those of cells exposed to BTZ and significantly higher than those of UV-, ONX0914- or RA190-induced apoptotic cells which were devoid of type I IFN signature (**Figures 4**, **5**). Whether the immunogenic effects of BTZ and/or PR619 is exclusively attributed to their capacity of inducing type I IFN genes remains unclear. However, the observation that ONX0914-induced cell death delivers canonical ICD signals (**Figure 7**) but fails to promote DC maturation (**Figure 5**) supports this assumption.

Altogether, our study identifies PR619 as a new member of the growing family of apoptosis-inducing agents causing ICD (52, 134). Whether the immunogenic potential of PR619 is due to a global disturbance of the cellular proteome or the de-ubiquitination of specific sets of proteins remains to be determined.

## Data availability statement

The raw data supporting the conclusions of this article will be made available by the authors, without undue reservation.

## Ethics statement

The studies involving human participants were reviewed and approved by the Universitätsklinikum Jena Review Board (Ethic number 2018-1157-Material), 07747 Jena, Germany. The patients/participants provided their written informed consent to participate in this study.

## Author contributions

ZW, RA-A, GÇ, JS, and FE performed the experiments. AB, HME, EK, and FE contributed to literature searches, designed the experiments, and analyzed data. FE wrote the manuscript and prepared the figures. All authors reviewed, edited, and approved the submitted version of the manuscript.
