# Supplementary material for: Immunogenic cell death triggered by impaired deubiquitination in multiple myeloma relies on dysregulated type I interferon signaling
Source: Front Immunol. 2023 Mar 2;14:982720. doi: 10.3389/fimmu.2023.982720 (PMC10018035; doi:10.3389/fimmu.2023.982720)
Supplement: Supplementary file 1 [file DataSheet_1.docx]

Immunogenic cell death triggered by impaired deubiquitination in multiple myeloma relies on dysregulated type I interferon signaling

**Zeinab Waad Sadiq, Annamaria Brioli, Ruba Al-Abdulla, Gonca Çetin, Jacqueline Schütt, Hugo Murua Escobar, Elke Krüger and Frédéric Ebstein**

**Supplemental figures:**

**Fig. S1**: Cell viability dose-response curves of seven MM cell lines to protein homeostasis disruptors.

**Fig. S2**: Proteasome subunit characterization of seven MM cell lines.

**Fig. S3**: Densitometry analysis of the expression levels of the standard (β1, β2, β5) and immunoproteasome subunits (β1i, β2i, β5i) in seven MM cell lines.

**Fig. S4**: Analysis of the IFN-stimulated gene (ISG) expression profile in seven MM cell lines exposed to BTZ, ONX0914, RA190 or PR619.

**Fig. S5**: Analysis of the IFN-stimulated gene (ISG) expression profile in two unrelated primary MM cell lines treated with PR619.

**Fig. S6**: Densitometry analysis of the protein expression levels of the major components of the UPR and ISR in NCI-H929 cells treated exposed to BTZ, ONX0914, RA190 or PR619.

**Fig. S7**: Western-blot and densitometry analysis of the expression protein levels of the UPR and ISR sensors in response to BTZ, ONX0914, RA190, PR619 as well as the ER stress inducer tunicamycin.

**Fig. S8**: Densitometry analysis of the STAT1, (p)STAT1, IRF3, (p)IRF3, TBK1 and (p)TBK1 protein expression levels in NCI-H929 cells pretreated with various signaling pathway inhibitors and subsequently incubated with PR-619.

**Fig. S9**: Effects of various signaling pathway inhibitors on MX1 gene expression induced by PR619 in NCI-H929 cells.

**Fig. S10**: Densitometry analysis of caspase-3 and cleaved caspase-3 steady-state protein expression levels in NCI-H929 cells in response to DMSO, PR619, BTZ, doxorubicin and UV-B exposure.

**Fig. S11**: Analysis of the IL-24 gene expression basal level in seven MM cells lines and in response to BTZ, ONX0914, RA190 and PR619 in NCI-H929 cells.

**
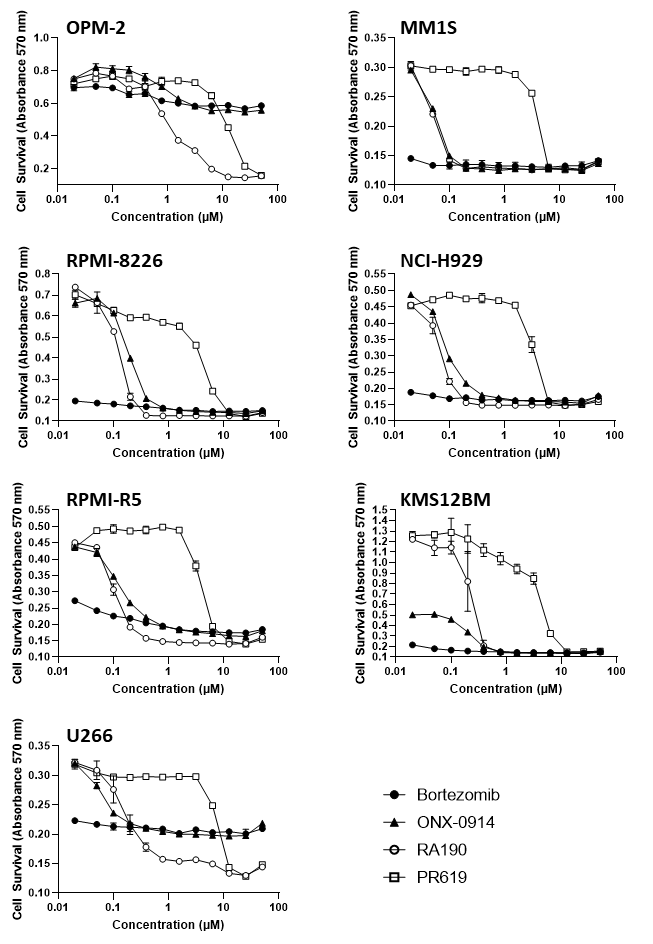
**

**Fig. S1: Cell viability dose-response curves of seven MM cell lines to protein homeostasis disruptors.** Cell survival of OPM-2, RPMI-8226, RPMI-R5, U266, MM1S, NCI-H929 and KMS12BM MM cell lines was determined after 24h by MTT (1 mg/mL) assay in the presence of increasing concentrations (50 nM-50 µM) of bortezomib (BTZ), ONX-0914, RA190 or PR619, as indicated. Data are shown as means ± standard error (SE).


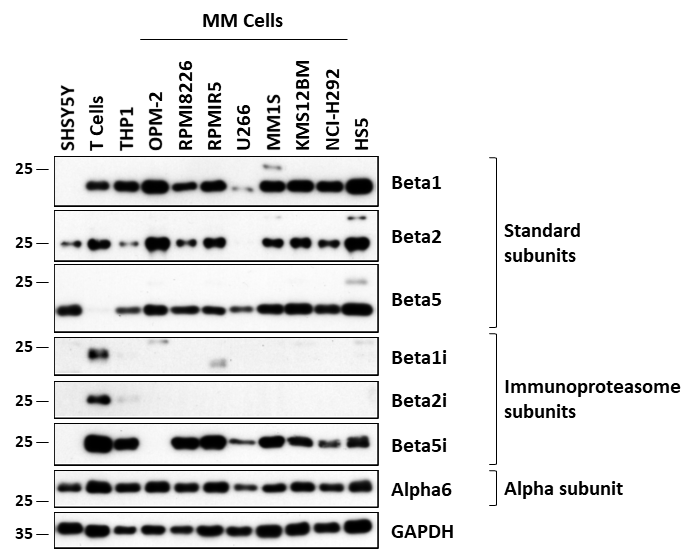


**Fig. S2: Proteasome subunit characterization of seven MM cell lines.** THP1, OPM-2, RPMI-8226, RPMI-R5, U266, MM1S, NCI-H929, KMS12BM MM and HS5 cell lines were subjected to protein extraction (RIPA lysis) prior to SDS-PAGE and western-blotting using antibodies specific for the standard proteasome subunits β1, β2 and β5, the immunoproteasome subunits β1i, β2i and β5i, as indicated. Controls for the standard and immunoproteasome subunits consisted of whole-cell lysates derived from SHSY5Y cells (first lane) and T cells (second lane), respectively. Equal protein loading was ensured by probing the membranes with an anti-GAPDH antibody.


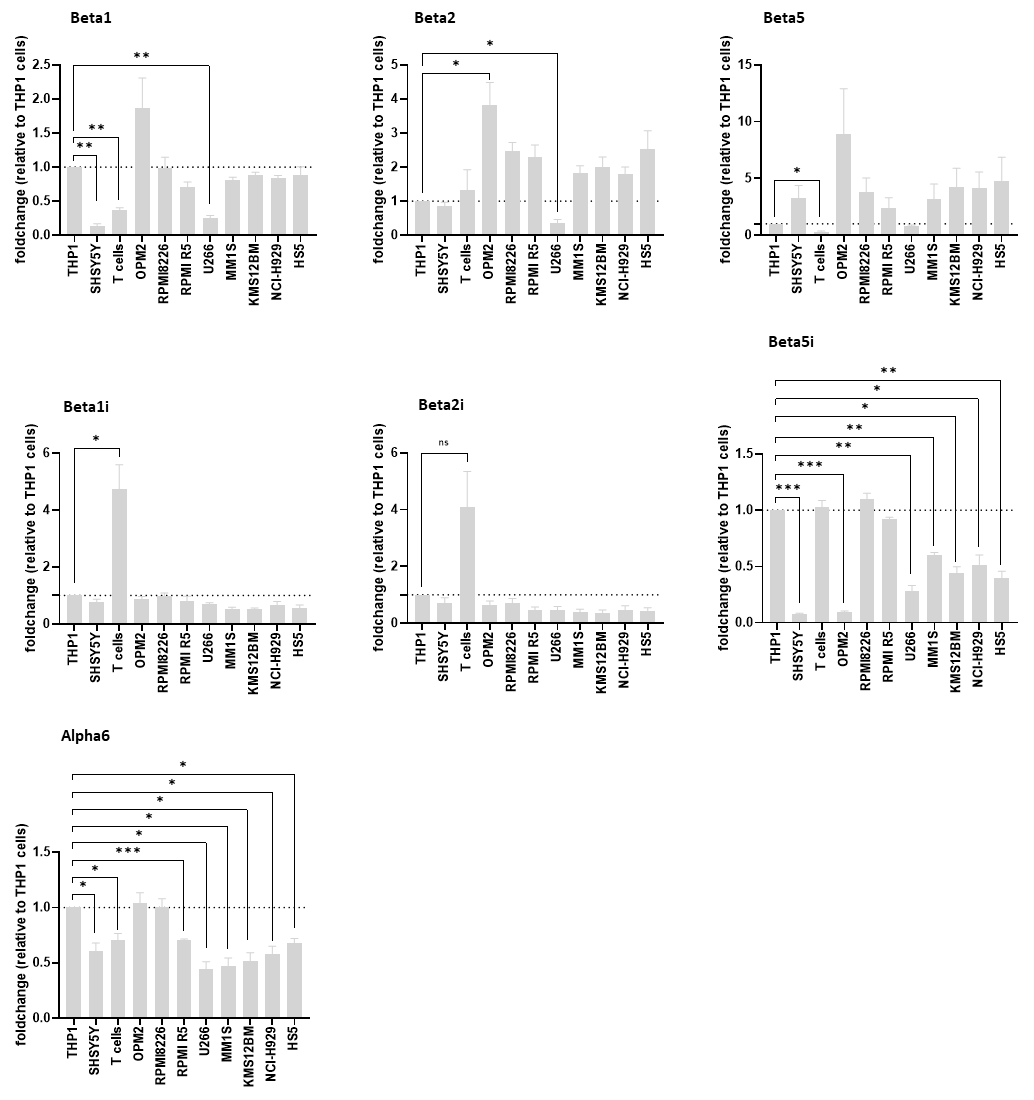


**Fig. S3: Densitometry analysis of the expression levels of the standard (β1, β2, β5) and immunoproteasome subunits (β1i, β2i, β5i) in seven MM cell lines.** Densitometry analysis showing the relative protein contents detected by western-blotting in MM cell lines (OPM-2, RPMI-8226, RPMI-R5, U266, MM1S, NCI-H929, KMS12BM) as well as THP1, SHSY5Y, HS5 and T cells, as indicated. The y-axis represents the fold change means ± standard error of the mean (SEM) in densitometry measurements from 3 independent experiments (n=3) which were set as 1 for THP1 cells. Statistical significance was assessed by paired t test (**p*<0.05, ** *p*<0.01, *** *p*<0.001), ns: not significant.


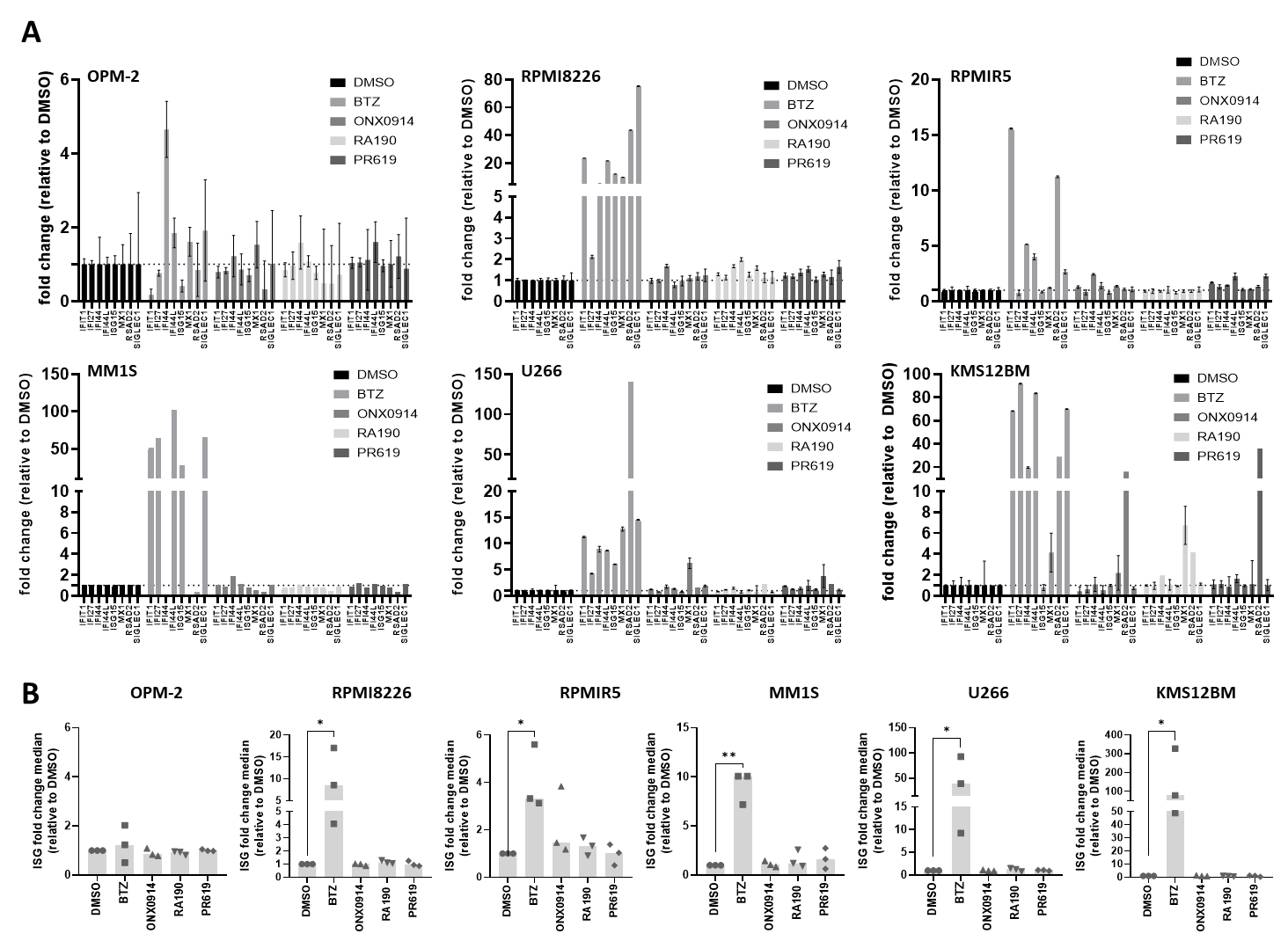


**Fig. S4: Analysis of the ISG expression profile in seven MM cell lines exposed to BTZ, ONX0914, RA190 or PR619.** A**.** Gene expression of eight typical IFN-stimulated genes (*IFI27*, *IFIT1*, *IFI44*, *IFI44L*, *ISG15*, *RSAD2*, *MX1* and *SIGLEC1*) was assayed by RT-qPCR on OPM-2, RPMI8226, RPMIR5, MM1S, NCI-H929, KMS12BM and U266 MM cell lines after a 12-h exposure to BTZ, ONX0914, RA190, PR619 or DMSO (control), as indicated. Expression levels were normalized to housekeeping genes and relative quantifications (RQ) are presented as fold change over cells exposed to DMSO. Shown is one representative experiment out of at least three. B. Shown are fold change median values of the eight ISG over DMSO measured in three independent experiments. Statistical significance was assessed by paired t test (**p*<0.05, ** *p*<0.01, *** *p*<0.001).


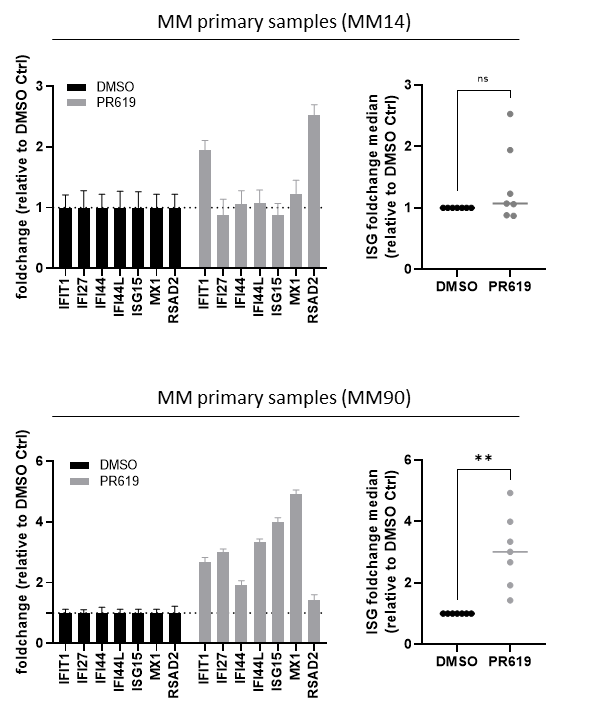


**Fig. S5: Analysis of the IFN-stimulated gene (ISG) expression profile in two unrelated primary MM samples treated with PR619.** Gene expression of seven IFN-stimulated genes (*IFIT1*, *IFI27*, *IFI44*, *IFI44L*, *ISG15*, *MX1* and *RSAD2*) was assayed by RT-qPCR on the two primary MM samples MM14 and MM90 isolated from relapsed patients, as indicated. Expression levels were normalized to GAPDH and relative quantifications (RQ) are presented as fold change over controls. Shown is also the median fold expression of the seven ISG over DMSO controls. Statistical significance was assessed by paired t test where ** indicates *p*<0.01, ns: not significant.


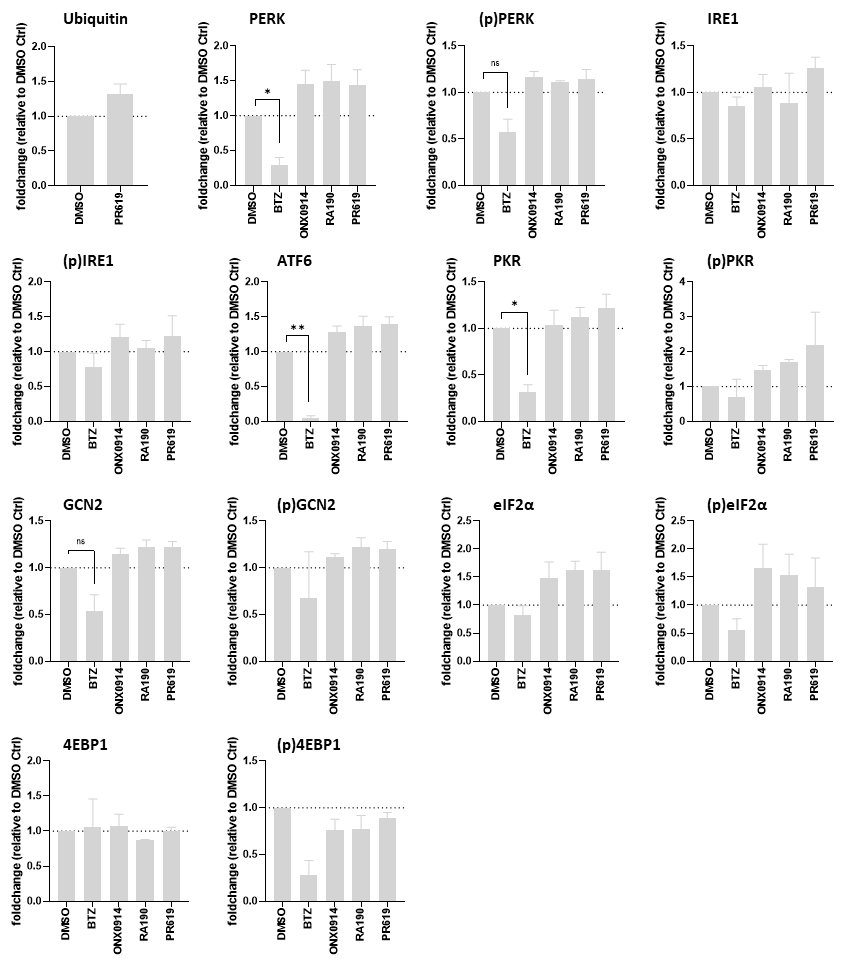


**Fig. S6: Densitometry analysis of the protein expression levels of the major components of the UPR and ISR in NCI-H929 cells treated exposed to BTZ, ONX0914, RA190 or PR619.** Densitometry analysis showing the relative protein contents of ubiquitin-protein conjugates; PERK, (p)PERK, IRE1, (p)IRE1, ATF6, PKR, (p)PKR; GCN2, (p)GCN2, eIF2α, (p)eIF2α, 4E-BP1 and (p)4E-BP1 detected by western-blotting in NCI-H929 cells in response to DMSO, BTZ, ONX0914 or PR619 treatments, as indicated. The y-axis represents the fold-change means ± standard error of the mean (SEM) in densitometry measurements from 3 independent experiments (n=3) which were set as 1 for DMSO. Statistical significance was assessed by paired t test (**p*<0.05, ** *p*<0.01), ns: not significant.


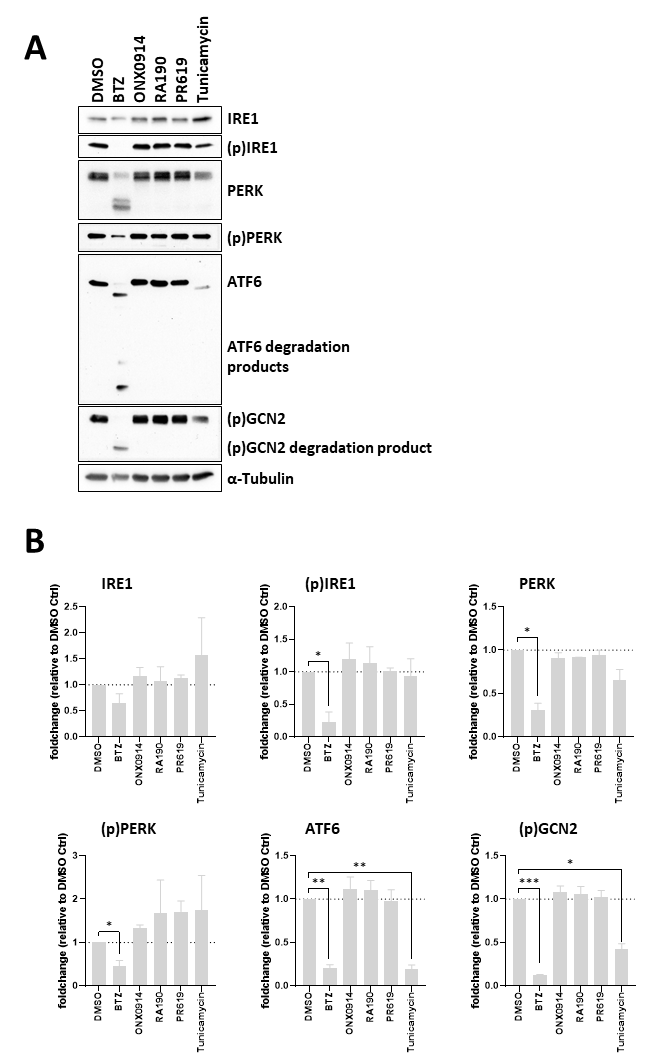


**Fig. S7: Western-blot and densitometry analysis of the expression protein levels of the UPR and ISR sensors in response to BTZ, ONX0914, RA190, PR619 as well as the ER stress inducer tunicamycin.** A. NCI-H929 cells exposed to DMSO, BTZ; ONX0914, RA190, PR619 or tunicamycin were subjected to protein extraction (RIPA lysis) prior to SDS-PAGE and western-blotting using antibodies specific for IRE1, (p)IRE1, PERK, (p)PERK, ATF6, (p)GCN2 and α-tubulin (loading control), as indicated. B. Densitometry analysis showing the relative protein contents of the samples described in A. The y-axis represents the fold-change means ± standard error of the mean (SEM) in densitometry measurements from 3 independent experiments (n=3) which were set as 1 for DMSO. Statistical significance was assessed by paired t test (**p*<0.05, ** *p*<0.01).


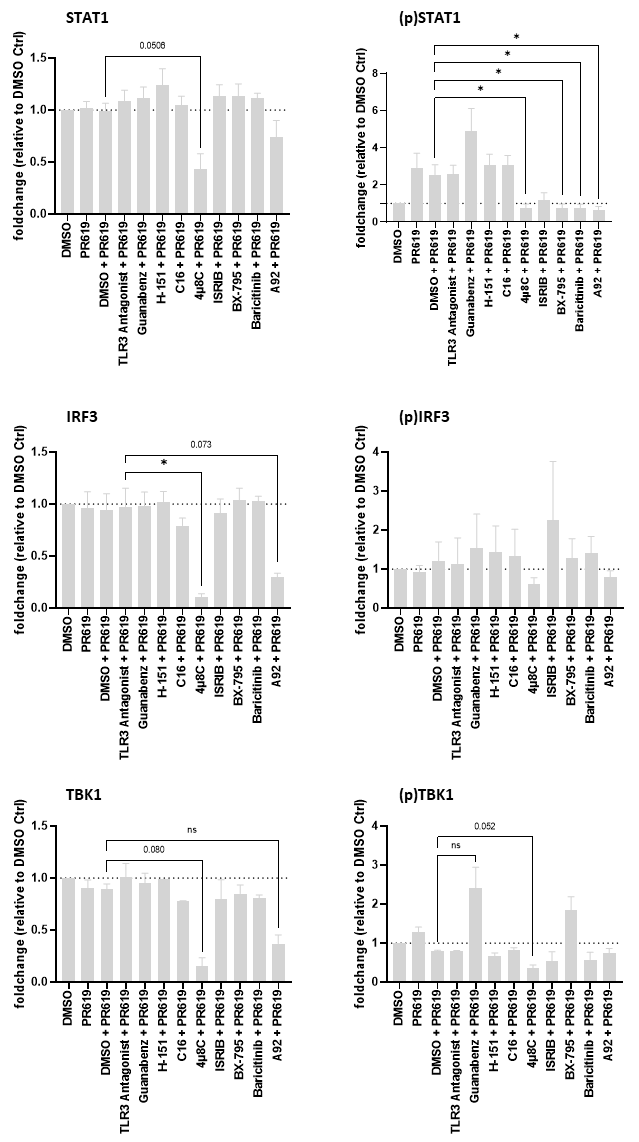


**Fig. S8: Densitometry analysis of the STAT1, (p)STAT1, IRF3, (p)IRF3, TBK1 and (p)TBK1 protein expression levels in NCI-H929 cells pretreated with various small-molecule inhibitors before incubation with PR-619**. Densitometry analysis showing the relative protein contents of STAT1, (p)STAT1, IRF3, (p)IRF3, TBK1 and (p)TBK1 in NCI-H929 cells in response to PR619 ± various inhibitors, as indicated. The y-axis represents the fold-change means ± standard error of the mean (SEM) in densitometry measurements from 3 independent experiments (n=3) which were set as 1 for DMSO. Statistical significance was assessed by paired t test (**p*<0.05), ns: not significant.


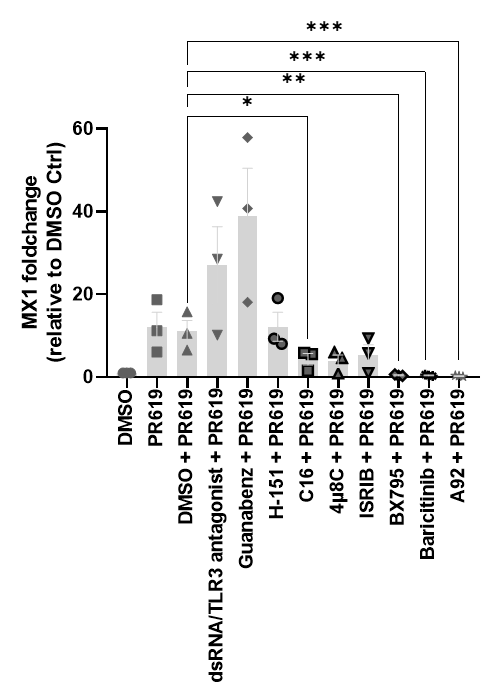


**Fig. S9: Effects of various signaling pathway inhibitors on MX1 gene expression induced by PR619 in NCI-H929 cells.** A. NCI-H929 cells were exposed to DMSO, TLR3/ds RNA antagonist (100 µM), guanabenz (50 µM), H-151 (2 µM), C16 (1 µM), 4µ8C (100 µM), ISRIB (200 nM), BX795 (1 µM), baricitinib (1 µM) or A92 (10 µM) for 2 hours prior to an overnight treatment with DMSO or PR619 (1,5 µM), as indicated. Samples were subjected to RNA extraction and subsequent RT-qPCR analysis for MX1 gene expression. Expression levels were normalized to housekeeping genes and relative quantifications (RQ) were carried out over cells exposed to DMSO. Shown are fold change median values of MX1 over DMSO from three independent experiments. Statistical significance was assessed by paired t test (**p*<0.05, ** *p*<0.01, *** *p*<0.001).


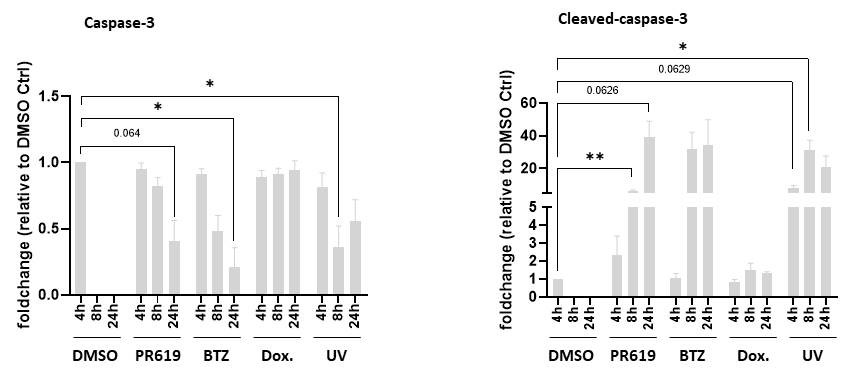


**Fig. S10: Densitometry analysis of caspase-3 and cleaved caspase-3 steady-state protein expression levels in NCI-H929 cells in response to DMSO, PR619, BTZ, doxorubicin and UV-B exposure.** Shown is the densitometry analysis showing of the relative protein contents of caspase-3 and cleaved caspase-3 in NCI-H929 cells following a 4, 8 and 24h-treatment with DMSO; PR619, BTZ doxorubicin (Dox.) or UV-b exposure, as indicated. The y-axis represents the fold-change means ± standard error of the mean (SEM) in densitometry measurements from 3 independent experiments (n=3) which were set as 1 for DMSO at 4h. Statistical significance was assessed by paired t test (**p*<0.05, ***p*<0.01).


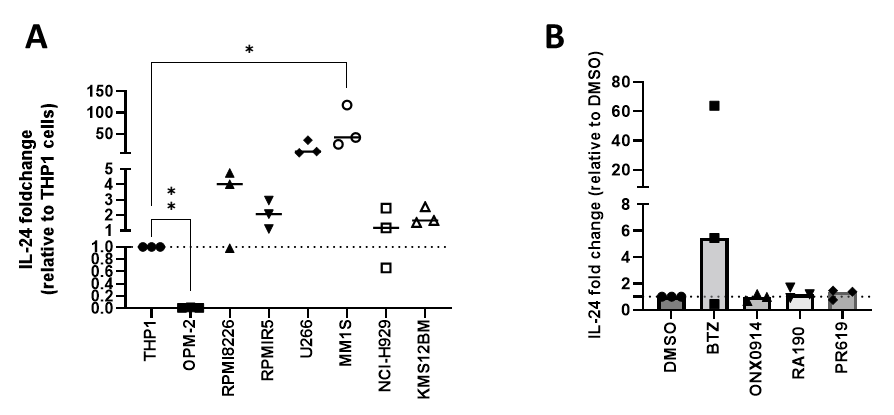


**Fig. S11: Analysis of the IL-24 gene expression basal level in seven MM cells lines and in response to BTZ, ONX0914, RA190 and PR619 in NCI-H929 cells.** A. Total RNA was extracted from the THP1, OPM-2, RPMI-8226, RPMI-R5, U266, MM1S, NCI-H929 and KMS12BM cell lines prior to cDNA synthesis and qPCR using primers specific for IL24. Expressions levels of *IL24* are shown as fold change median values over the THP1 cell line from three independent experiments. Statistical significance was assessed by ratio paired t test (**p*<0.05, ***p*<0.01). B. NCI-H929 cells exposed to DMSO, BTZ, ONX0914, RA190 or PR619 were assessed for their content of IL24 transcripts by RT-qPCR. Shown are IL24 fold changes over DMSO controls.
